# State Examinations for Medical License in Pennsylvania

**Published:** 1903-08

**Authors:** 


### State Examinations for Medical License in Pennsylvania

AT the recent meeting of the Medical Society of the State of Pennsylvania, Dr. O. A. J. Kelly, in an address on medicine, leveled a criticism at the Pennsylvania State Board of Medical Examiners, totally unjustifiable and wide of the real facts. Dr. Kelly must have been misinformed, or he would not have made such an unusual blunder as is shown by the paper which we print below from the pen of Dr. T. D. Davis, and taken from the June issue of the *Pennsylvania Medical Journal*.

Dr. Davis is vice-president of the American Academy of Medicine, is a physician of great experience and high standing, and appears to be quite familiar with state examinations for medical license. The paper is important, because it bears upon the general questions of state license in all the states; hence, we take pleasure in republishing it. The editor of the journal from which it is taken, says it is published "by request," but no apology is needed for printing such an able paper.

### THE RECENT ADDRESS IN MEDICINE AT OUR STATE MEDICAL SOCIETY.[1]

By THOMAS D. DAVIS, M.D., Pittsburg, Pa.

There is no subject so vital to the medical profession, at present, as its educational requirements. Closely allied to this is the organisation of the medical practitioners into a profession. Without this organisation there is no such thing as The Medical Profession. The supreme courts have ruled, "That any man is a doctor who calls himself such," and that, "Legally one doctor is as good as another." Medical societies of more or less importance, for scientific purposes, have existed here and there for many years, but a real effort to combine medical practitioners into an organised profession, that shall be recognised as such, by the people and the laws, has only been attempted during this generation. It has been almost entirely through this organised profession, that the recent great advances in educational requirements have been secured. For the very first difficulty that impeded this effort at organisation was the diversity of educational qualifications of practitioners. To overcome this obstacle our medical schools were importuned to elevate and make uniform their requirements for entrance and graduation. The Association of Medical Colleges accomplished something in this line, but not until the establishing of the State Medical Examining Boards was this demand felt by all schools to be absolutely necessary.

---

1. Read in the Beaver County Medical Society, April 9, 1903.

The establishing of these boards in Pennsylvania ten years since was entirely the work of the medical society of the state, through its appointed committee, and was accomplished after more than four years of arduous work and against great opposition. That they have accomplished much good is beyond question. It is not correct to say that "they were coincident with improved and lengthened medical courses," for they were forerunners of this advance. So, in the future, as these boards require better preliminary and better professional education, will the colleges require it also. Of course, these boards are not perfect, and proper criticism is always in order. However, the attack on our own state board by Dr. Kelly, in his address on Medicine (?), at the Allentown meeting of The Medical Society of the State of Pennsylvania, seems to me is not meant for friendly criticism. It simply ridicules, is uncharitable and appears vicious. It certainly can only be excused on account of prejudice, or lack of due consideration.

Not to stop to criticise the good form of abusing a child in the home of its mother, Dr. Kelly certainly has not read the act that established these examining boards in our state. In no place is it stated or implied that the act is, "To maintain the honor and reputation of the profession, . . . to prevent the charlatan and mountebank from humbugging." Quacks we have and humbugs also and possibly always will have them, but if possible let them be not entirely ignorant.

The reason for the establishing of these boards is, "That none but competent and properly qualified physicians and surgeons shall be allowed to practise their profession," and if possible bring all up to an educational standing of at least minimum requirements. It would be a consummation devoutly to be wished to have some judicatory that should pass on their moral qualifications also, but that is chimerical and only can they be reached by law when their acts become indictable. I wish our boards had the power Dr. Kelly seems to think they have. However, the address may not be intended to apply to our state alone; indeed it cannot be, for he claims that the medical practice laws were especially planned to control the Eddyite, the Dowieite, the christian scientist and osteopath, when all of these fakes have originated or come into prominence since our state medical examining boards were established, and this is true concerning a number of other states also. But even in the states that have more recent medical laws, not one word is aimed against any one in particular, but simply that all who practise medicine may at least have an educational qualification, and owing to the numerous and poorly equipped medical schools, this qualification must not rest on a diploma alone.

Dr. Kelly is surely defective in his history when he minimises the influence of these laws, for when an examining board was first urged by the Medical Society of the State of Pennsylvania, there was not a medical college in the state, except the Hahnemann, that had more than a two years' course; indeed, they had only a one year's course, but two years' attendance was required for graduation, and when the boards were finally established, no regular college in the state had more than a three years' course—if that.

The address jumps with such nimbleness from one theme to another, that it is impossible to follow it closely, but I wish to consider its reflection on the State Medical Examining Boards and its views concerning "reciprocity." This may not be necessary in this presence, for he simply voices what has been suggested by others, and has been already answered. I presume, of course, as the address was delivered before and by the appointment of the Medical Society of the State of Pennsylvania, its strictures refer more particularly to the boards of our own state. First, he acknowledges some good accomplished by these boards, yet the praise seems grudgingly given and "radical changes are imperative." Next, he makes an unwarranted and unsupported attack on the members of the board. He claims in choice and gracious terms that they have, " a woeful lack of knowledge;" "manifest a disposition to be unfair, and an absence of judicious discrimination;" "are incapable of formulating a question in correct English, or one that is not ambiguous, unfair, inane, or asinine;" that some of the examiners, "are absolutely heedless of the progress of medicine;" "are more familiar with practical politics than the present state of medical science; and "have not added a book to their libraries for thirty years." If these accusations are all true, (but Dr. Kelly forgot to give a scintilla of proof to sustain them), then it follows, as none of these examiners ever passed a medical examining board, but are the pure product of the colleges before such boards were established they therefore furnish positive proof of the absolute necessity of these boards, and that the colleges cannot be trusted to produce qualified practitioners. Q. E. D.! But is that what the doctor intended to prove?

Having thus paid his respects to his fellow members of the state society on these boards, he proceeds to characterise the questions they ask as asinine! Now, as far as I can find recorded the only ass that ever spoke asked very fine questions! After hunting over the many hundred questions asked by the Pennsylvania Board, he cannot find enough bad ones to suit his purpose and so borrows some from other states, (with the covert purpose, I presume, of establishing reciprocity), and because he cannot get enough bad ones there he boldly crosses the ocean to Merry Old England and imports a few more! It is not my intention to defend poor questions from whatever source they may originate, but does Dr. Kelly know that in Pennsylvania and New York, at least, the medical examiners do not formulate the questions they have to ask? Or by what process of "active cerebral matter" that is not entirely destitute of logical cells does Dr. Kelly arrive at the conclusion, "that bad spelling, bad grammer, bad capitalisation, bad punctuation, the incorrect use of words, etc., by the student, is a reflection on the examining board asking the questions?" Does he mean, that if the examinations were oral these defects could not be detected and therefore the board is to blame? From another part of the address I should judge this is what he does mean, otherwise there seems to be no possible sense in the statement.

The art of asking educational questions is a pedagogical function of acknowledged difficulty, and characterises only the best teachers. It is not, therefore, a wonder that some very poor questions are occasionally asked by these boards, but having looked over a large number I am surprised at their general excellence. It is the opinion of several of my young medical friends who have taken both examinations, that those of the state board are uniformly fair and not so difficult as those for competitive appointments to most hospitals.

Dr. Kelly errs again, it seems to me, when he insists that these questions should be entirely on practical points. It is the fundamental principles that are the essentials. Almost any quack or nurse can give odds to a recent graduate on practical points. They know what to do, but not why. The office students of the old practitioner used to learn how and what to do, but they often became mere empirics. The sole argument for the existence of medical colleges is to teach the fundamentals and build a scientific foundation. If this is not their chief function, then doctors' offices and hospitals are incomparably better for the embryo doctor and the student sinks into the apprentice. The utilitarian idea is running wild in some quarters. A student gets his practical knowledge at the clinics and his fundamental knowledge in the class room, and it is with the class room that the state boards are most concerned, for their chief object must be to require this essential knowledge; the practical knowledge will come later and for it the student will be examined by the great public.

He also sneers at the minutiæ taught in the class room and, if he is correct concerning how soon it is forgotten, then it is a base deception and a fraud to compel a student to attend four years' medical courses to learn such stuff. Dr. Kelly wields a two-edged sword, that cuts which ever way it is turned.

Again, the address objects to written examinations, because they are of no use, but "to prove a good or bad memory." Surely as already noticed, they show the applicant's scholarship and preliminary training. I have tried in vain to find out just what Dr. Kelly does want. I am utterly unable to reconcile the assertion that "written examinations by *no means* achieve the ends for which they were instituted," with his statement, that written examinations, "to some extent, *at least,* should be continued, must be conceded." Such shifting certainly shows very active cerebral matter! However, he urges what Dr. Weir Mitchell has suggested, that applicants be required to diagnosticate and treat real disease in the living patient and perform operations on the cadaver. From one who especially advocates "practical" ideas this is an astonishing proposition. I hardly need take your time to show the absurdity and impracticability of this suggestion. Even if exactly the same kind of patients, enough cadavers and a suitable place could be secured, and then the necessary time taken for such an examination of three hundred or more applicants, who would decide whether, "the examinee had furnished evidence of his practical ability? "Surely not those members of the board already so considerately described. But even if Dr. Kelly himself and others were appointed, there is always room for a wide difference of opinion on diagnosis, treatment and operations, and where doctors differ who would decide? No, in all such examinations we must select the subjects on which there is the most unanimity of opinion and the least room for honest differences.

To use the figure in the address, it is the solid foundation of all medical knowledge, the part that is below the ground that we want the expert to examine; the superstructure is visible, is inquired into, is examined, is put to practical uses and is criticised,—by the public. However, the greatest objections, to oral examinations, and which cannot be overcome, are that they give room for partiality, even fraud, are not available for comparison, and cannot be put on record for reference, or if needs be for evidence. It is also more than possible that the oral method may account for so many ignorant students passing the medical college examinations; at least, where written examinations were formerly unknown, they are now being used more or less by all colleges. Whether the "certificates of the state medical examining boards be a source of pride to those who secure them," or not, it is a fact that a certain 20 per cent. of those who are graduated by the medical schools would be rejoiced to have them!

Under so-called reciprocity, the address has nothing to offer that is not already being tried. It is in error again, however, when it states, "That a man may be a doctor in one state and not in another . . . a condition absolutely anomalous, and without its counter part in the practice of any other profession." Surely Dr. Kelly knows that a man may be a lawyer in one county even and not in another. Let a Philadelphia lawyer try to be admitted to the Lancaster county bar and see what difficulty he will have, or let him cross the Delaware river and he will find he has to reside three full years in New Jersey before he can become a full-fledged lawyer in that state. So in many religious denominations, going from one jurisdiction to another, a minister is compelled to stand an examination which may extend even to his college studies.

It seems to me, under the present conditions of development of our country, the easy transfer of a physician from one state to another would defeat all efforts to advance medical education. Scores of doctors who have been refused certificates by the Pennsylvania boards are now practising medicine in other states. On the other hand, in sparsely settled and poor parts of our country, where compensation is very small and diseases more simple, a doctor should not be required, (although greatly to be desired) to make the expensive preparation necessary in the older states, and such is the case. Now if such doctor should remove to a state with a higher educational standard, it is only fair that he shall prove his qualifications.

Two grades of examinations, as the address suggests, would be manifestly unfair. For, if a number of doctors have passed the higher and harder grade, it would surely be an injustice to them, to allow one to come in and practise beside them who has only passed the easier grade, simply because perchance he has practised honorably in a state that has no requirements at all. Beside, I doubt very much, if any legislature would pass a law giving power to a board of medical men to decide, "who was an honorable practitioner of medicine." That is the function of the courts.

I realise that to some worthy men it works a hardship, but these are comparatively few and mostly make the change to better their pecuniary condition; but, I submit, these few, thoroughly qualified, honorable men, ought to be willing to suffer to keep out the hundreds, yea thousands of peripatetic fellows, who mostly change locations on account of their ignorant impositions.

To those who have had no experience with legislative bodies, it seems as if it would be an easy matter to have laws passed or amended, especially if they were intended for the public good, but such is far from the case. There are two sides to every question. All do not see alike. What some doctors may think is for the public good, legislators may think is positively pernicious. Attempting to amend laws is sometimes like opening Pandora's box. No medical legislation should be attempted until carefully considered and authorised by the organised profession of the state or nation. I think the recent attempt to amend the medical laws of this state, was untimely and is likely to result in much more harm than good. The examining boards have more power now than they exercise. Let them educate the profession and the people up to the full scholarly requirements that they can demand now, before asking for more power. There is absolutely no need and it is an imposition to require a student to spend four years in a medical college with the present curriculum. If the medical colleges will make their courses so hard that it will require four years to master it, that is another question, but as at present conducted, three years in a college and one in a hospital is far better. What the state board should demand is, that the applicant *has the knowledge,* and not where and how long it took him to get it. It might be possible for a student to get more and better knowledge from a private tutor and in a hospital! Let the boards put their educational requirements so high that it will take four or even more years in our medical colleges as at present conducted to attain them. However, if some one is particularly bright, or can acquire the same amount of knowledge in less time, possibly on account of previous training, or by rigid application, why not let him do so? The law should not hold back, but encourage. Ability, application and preliminary training are of far more importance than the mere time consumed in a medical college, and these can be secured by the board by the severity of their examinations.

Our state medical examining board is endeavoring to advance and unify medical education. Let us trust it. Let us encourage, advise and assist, but throw no impediment in its way. Let us study to advance in the light of the past, realising out of what depths our profession has emerged. Rome was not built in a day, and we must not be impatient. Vast strides have already been taken. Let us criticise kindly, yet firmly, and advance if slowly, yet surely. The burden of the profession will soon be on the shoulders of those who have passed the state examining boards, and to them we will bequeath a better educated and a more united profession than was ever thought possible at the beginning of our generation. There is still much to be accomplished. We must get closer and closer together as a profession. While whole counties in our own state have no medical organisations and more than four thousand regular practitioners are outside of the organisation in this state, let us feel our shortcomings and do nothing to foment discord, but become zealous to unify our noble calling into a great and harmonious profession.

